# Modeling the Effect of High Calorie Diet on the Interplay between Adipose Tissue, Inflammation, and Diabetes

**DOI:** 10.1155/2019/7525834

**Published:** 2019-02-03

**Authors:** V. Prana, P. Tieri, M. C. Palumbo, E. Mancini, F. Castiglione

**Affiliations:** ^1^Institute for Applied Computing (IAC) “M. Picone”, National Research Council of Italy (CNR), Via dei Taurini, 19-00185 Rome, Italy; ^2^Institute for Advanced Study (IAS), University of Amsterdam (UvA), Oude Turfmarkt, 147-1012 GC Amsterdam, Netherlands

## Abstract

**Background:**

Type 2 diabetes (T2D) is a chronic metabolic disease potentially leading to serious widespread tissue damage. Human organism develops T2D when the glucose-insulin control is broken for reasons that are not fully understood but have been demonstrated to be linked to the emergence of a chronic inflammation. Indeed such low-level chronic inflammation affects the pancreatic production of insulin and triggers the development of insulin resistance, eventually leading to an impaired control of the blood glucose concentration. On the contrary, it is well-known that obesity and inflammation are strongly correlated.

**Aim:**

In this study, we investigate *in silico* the effect of overfeeding on the adipose tissue and the consequent set up of an inflammatory state. We model the emergence of the inflammation as the result of adipose mass increase which, in turn, is a direct consequence of a prolonged excess of high calorie intake.

**Results:**

The model reproduces the fat accumulation due to excessive caloric intake observed in two clinical studies. Moreover, while showing consistent weight gains over long periods of time, it reveals a drift of the macrophage population toward the proinflammatory phenotype, thus confirming its association with fatness.

## 1. Introduction

Diabetes is a chronic disease characterized by a decreased production of insulin and by a reduced efficacy of the insulin produced. This impaired condition is differently caused by both type 1 and type 2 diabetes. In type 1 diabetes, insulin-producing cells in the pancreas (*i.e.*, the beta cells) have been damaged by the immune system in a *de facto* autoimmune response. In type 2 diabetes, the origin of beta cell malfunctioning is diverse and mainly attributed to a systemic low-grade inflammation which also impairs the various organs ability to make use of insulin and remove glucose from the blood.

If untreated, both forms of diabetes result over time in a persistent high concentration of glucose in blood which leads to serious tissue damage, especially to the nervous and circulatory system with potentially fatal consequences. In 2015, diabetes caused five million deaths worldwide. The number of diabetic individuals has risen over the years from 285 million in 2010 to 387 million in 2014 and 415 in 2015, while projections estimate 642 million in 2040 [1].

There is no doubt that this kind of “pandemic” requires maximum attention and that besides clinical and biological research, mathematics could contribute to shed light on this complex pathology. In our study, we employed computational modeling and simulation to describe the effects of high calorie diets on the pathology of type 2 diabetes limiting our observation to the process of weight gain ultimately leading to the onset of an inflammation state.

### 1.1. About Type 2 Diabetes

Symptoms of type 2 diabetes (T2D) are not very pronounced, and as a result, the disease is usually diagnosed several years after its onset, once complications have already become established. The International Diabetes Federation estimated that 193 million people with diabetes are undiagnosed and are therefore at risk of developing complications. Since T2D comprises 90% of diabetes cases worldwide, by 2040, more than 570 million people will likely be living with diabetes, the majority of them being unaware of it [2]. Another cause of concern is the fact that while T2D was previously prognosticated only in adults, it is now also found in children [3]. It is also worth to be mentioned that T2D is becoming worrying in developing countries which have recently taken up higher calorie diets. For all these reasons, there is an urgent need to understand the complex mechanisms underpinning the onset of T2D and to identify early diagnostic parameters and related inflammatory indicators, possibly using a personalized medicine approach.

A number of conceivable stress mechanisms (herein indicated as “stressors”) leading to and participating in insulin resistance and beta-cell dysfunction have been hypothesized to explain the complex landscape of T2D onset, such as oxidative stress, endoplasmic reticulum stress, amyloid (*i.e.*, insoluble fibrous proteins) deposition in the pancreas, and ectopic lipid deposition in the muscle, liver, and pancreas [4]. All of these stressors are potentially linked to overnutrition although it has been difficult, so far, to identify the precise mechanisms determining the decreased rate of glucose uptake by the different tissues in individuals proceeding to T2D. However, it is noteworthy that each of these cellular stressors is thought to also either induce an inflammatory response by itself or to be exacerbated by or associated with inflammation [5]. Inflammation is a complex, systemic, multiscale physiological process necessary to cope with damaging agents, involving a variety of cells, organs, and organ systems. The complexity of the inflammatory process escapes reductionist approaches, since it is characterized, among other things, by nonlinear kinetics as well as numerous and nested feedback loops.

The ultimate conceptualization identifies the hallmark of T2D in a chronic inflammatory state initiated by an excess of nutrients and referred to as metabolic inflammation or *metaflammation*. Indeed, proof-of-concept clinical studies demonstrated the potential of using an anti-inflammatory molecule in T2D therapy, thus strongly linking inflammation with the pathogenesis of T2D [4].

This clear evidence is at the core of the present computer simulation study as it constitutes the primary working hypothesis of a mathematical model developed and used to assess the risk of developing an inflammatory state thus leading to T2D.

## 2. Computational Modeling

We used an agent-based model (ABM) of the immune system to study *in silico* the emergence of a low-level chronic inflammation in the adipose tissue, as the main place of accrual of inflammatory evidences due to prolonged excessive calorie intake.

The model is a derivation of a well-established general-purpose immune system simulator [6], a modeling framework that has been used over the past two decades to study different human pathologies [7–9], specific aspects of the immune response [10, 11], and also nonhuman immunity [12].

It is a multiscale discrete-event model, generic enough to account for the major hallmarks of the immune response. This computational model has been conceived to allow the dynamic representation of hypotheses and their preliminary testing [13]. The model represents several primary and secondary immune compartments playing a critical role in the immune response: generic tissue (e.g., epithelial and adipose tissue), lymphoid tissue, thymus gland, and bone marrow. These components are bundled together in a complex yet parsimonious model of immunity focused at the mesoscopic (i.e., cellular) level.

Agent-based modeling is based on a general paradigm for complex systems inspired by von Neumann's “cellular automata” [14]. It consists of discrete dimensional entities (three-dimensional space and time in discrete steps), where the agents are the relevant entities (cells or molecules) equipped with virtual receptors and capabilities, which reflect biological observations.

In the used modeling framework, key immune cells such as monocytes-derived macrophages, dendritic cells, and B and T lymphocytes are represented by agents and follow a set of rules describing the different phases of the recognition and response of the immune system against a pathogen. In particular, the model incorporates key immune processes such as phagocytosis, antigen presentation, cytokine release, cell activation from inactive or anergic states, development of the immune memory, cytotoxicity, and antibody secretion [6]. Additionally, this model reproduces the gene-regulation mechanisms leading to macrophage differentiation during the different stages of inflammation [11].

The model represents a small volume of the body populated by a fraction of immune cells which is calculated according to generic leukocyte formulas. The discrete time step is equivalent to eight hours of real life.

For the work here presented, the above described model has been enriched for the ongoing purpose of studying the effects of excessive caloric intake by including specific mechanisms pertaining the (i) accumulation of fat in the adipose tissue, (ii) consequential growth of the adipose tissue, (iii) the generation of proinflammatory cytokines, and (iv) the endmost polarization of key innate immune mediators such as the macrophages into the proinflammatory phenotype establishing *de facto* a low-grade inflammatory state. This implementation is described in the following section.

### 2.1. Modeling the Effect of Excess Calories on Adipocytes

Among the main risk factors of T2D is an excess in body weight which is often the result of fat accumulation over an extended period of time. This process is the result of an excess of caloric intake which is not balanced by a comparable caloric consumption due, for instance, to physical activity.

In the literature, there are very few attempts to quantify and model the link between the effects of high caloric intake [15–18] and the emergence of the inflammation state. In the present study, therefore, we present a model on the effects of food intake linked to a model of weight gain eventually driving a systemic low-grade inflammation. Our model represents besides key immune cells responsible for the inflammation process, a population of adipocytes as the sum of individual cells acting as reservoirs for fat and originators of inflammatory signals. Model's anthropometric inputs include the subject initial body weight (BW) in kilograms, height (H) in meters, age (A) in years, and gender (G) male or female. Starting from these parameters, the relationship between adipocyte diameter and body mass index (BMI) is determined as in equation (1) to set the initial distribution of the volume of adipocytes. This relationship has been estimated in [19] from data on 54 men and 207 women and links the diameter of an adipocyte of the *omental* adipose tissue (*ϕ*) with the BMI:

(1) $$\begin{array}{c} \phi \left(\text{BMI}\right)=\phi_{\text{c}}-\lambda e^{-\delta \text{BMI}}, \end{array}$$

where, respectively, for female and male, *ϕ*_c_=123 and 120  *μ*m, *λ*=198.445 and 74.905 *μ*m, and *δ*=0.061 and 0.049 m^2^/kg [19]. These values for the parameter *ϕ*_c_ for male and female are in accordance with another experimental finding [18], which shows a maximal cell diameter value ranging from 120 to 130 *μ*m. Omental fat constitutes the larger part of visceral adipose tissue (VAT, i.e., the main spot of the inflammation), and hence, we surmise that relation in equation (1) is valid for all adipocytes in VAT. Moreover, since the body mass index is calculated as BMI=BW/*H* ^2^, the relationship in equation (1) links the average diameter of a visceral adipocyte *ϕ* to BW and therefore it allows the estimation of the distribution of the initial diameter of the adipocytes making up the fraction of the adipose tissue given the BMI of the subject at the beginning of the simulated period.

Another value of use to set the initial condition of the model is the initial value of the fat mass FM(0). To this purpose, the following compartmental relation between fat and body weight [20] allows to compute FM as a function of the body weight and the free fat mass (FFM):

(2) $$\begin{array}{c} \text{FM}\left(\text{BW}\right)=\text{BW}-\text{FFM}\left(\text{BW};A,H\right). \end{array}$$

According to Westerterp's regression equations in [21], different values of FFM for men and women can be estimated on the basis of the age *A*, body weight BW, and height *H* as follows:

(3) $$\begin{array}{c} \text{FFM}\left(\text{BW};A,H\right)=\alpha +\beta A+\gamma H+\delta \text{BW}, \end{array}$$

where the parameters *α*, *β*, *γ*, *δ* depend on the gender and are, respectively, *α*=−12.47 kg, *β*=−0.074 kg/years, *γ*=27.392 kg/m, and *δ*=0.218 for female and *α*=−18.36 kg, *β*=−0.105 kg/years, *γ*=34.009 kg/m, and *δ*=0.292 for male.

During periods of excessive calorie intake, adipocytes grow in order to stock the energy surplus (*swelling*). The growth continues until a *critical* size for the diameter of a visceral adipocyte (*ϕ*_c_) is reached (equation (1)). Beyond this value, the adipocyte stimulates the generation (*adipogenesis*) of new adipocytes from precursor cells [15–17].

**Figure 1:**
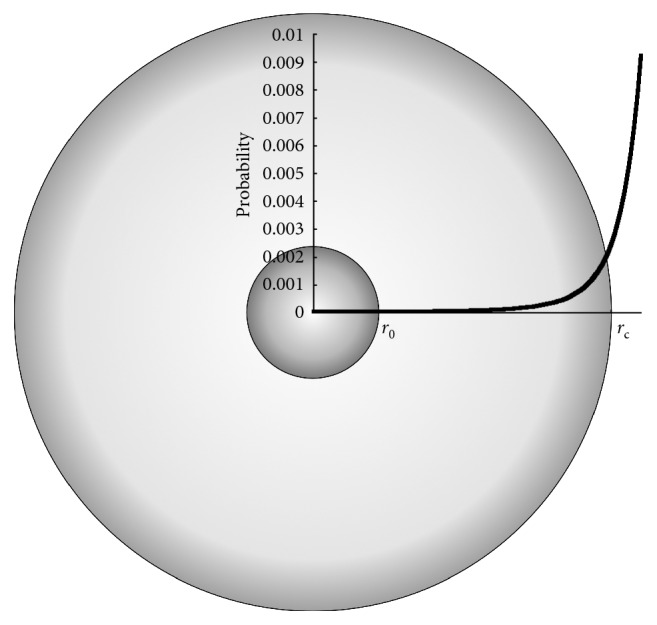
The volume of an adipocyte changes with an excess (or defect) of caloric intake. We model the *swelling* and also the recruitment of new adipocytes as stochastic events whose probabilities, respectively, *p*_s_ and *p*_a_ are function of the actual volume as in equation (5) and equation (8) and reach 0 and the maximum value, respectively, for $\underset{r⟶\phi_{c}/2}{\text{lim}}v\left(t\right)=v_{\text{c}}$ (Figure 2).

To model the enlargement of the adipocyte due to a (daily) surplus of calorie intake indicated by *E*, we assume a spherical shape and define the critical value for the volume for the cells *v*_c_=(4/3)*π*(*ϕ*_*c*_/2)^3^ where, as already mentioned above, *ϕ*_c_ is set to 123 *μ*m for females and 120 *μ*m for males [18, 19] (Figure 1).

We now define the *swelling factor* as the relation between the actual volume *v*(*t*) and the critical value *v*_c_:

(4) $$\begin{array}{c} \vartheta \left(t\right)=1-\frac{v\left(t\right)}{v_{\text{c}}}. \end{array}$$

Since we are representing cells as agents, we can model the increase of the volume of each single adipocyte individually. At each time step, the volume of an adipocyte changes with probability

(5) $$\begin{array}{c} p_{\text{s}}=\text{Pr}\left[\text{swelling}\right]=\frac{1}{2}\left[1+\tanh\left(k_{1}\vartheta \left(t\right)+k_{2}\right)\right], \end{array}$$

in view of the fact that larger cells are less likely to increment their volume than smaller ones. With this probability *p*_s_, the volume variation of each single adipocyte is described as follows:

(6) $$\begin{array}{c} v\left(t+1\right)-v\left(t\right)=\left\{\begin{array}{cc} k_{3}E+\eta , & \text{if }v_{0}\leq v\left(t\right)+k_{3}E+\eta ,\\ 0, & \text{otherwise,} \end{array}\right. \end{array}$$

where *η* ~ *𝒩*(0, *k*_4_ *v*_0_) is a Gaussian noise with zero mean and variance proportional to the *baseline volume* of an adipocyte *v*_0_=8.181 · 10^−6^ *μ*L calculated for a “normal” individual (approximate value) [22, 23].

Note that in equation (6), the volume *v*(*t*) is not allowed to become smaller than *v*_0_ even in case of successive negative values of *η* and *E* (as for a prolonged low caloric intake diet, *i.e.*, *E* < 0 kcal/day). Also, note that even though equation (6) does not impose constrains on the growth of the volume, the probability *p*_s_ becomes negligible when *v*(*t*) approaches 2*v*_c_ and hence the volume of the adipocytes cannot become too large.

To model the adipocyte recruitment from precursors at each step of the simulation, we first define the fraction *ζ*(*t*) of adipocytes which, by enlargement due to caloric excess intake, have surpassed the critical value *v*_c_ and are thus secreting growth factors (*i.e.*, adipokines) signaling adipocyte precursors for the need to generate new cells [16, 17, 26], that is,

(7) $$\begin{array}{c} \zeta \left(t\right)=\frac{\left|\left\{\text{cell }i:v_{i}\left(t\right)>v_{\text{c}}\right\}\right|}{N\left(t\right)}, \end{array}$$

where *N*(*t*) is the total number of adipocytes in the simulated volume. Then with probability *p*_a_ defined below, we generate new adipocytes at a rate of one cell/time step per microliter of adipose tissue volume:

(8) $$\begin{array}{c} p_{\text{a}}=\text{Pr}\left[\text{adipogenesis}\right]=\frac{1}{2}\left[\tanh\left(k_{5}\zeta \left(t\right)+k_{6}\right)+\tanh\left(1\right)\right]. \end{array}$$

The newly generated adipocytes increase the total adipose tissue volume since each adipocyte is initialized with volume *v*_0_. It is worth to note that, in case of prolonged excess of calories, this new tissue amounts to a new reservoir for further accumulation of fat, whereas if the diet is low calorie (data not shown), the weight is initially lost relatively quickly because of the shrinkage of the adipocytes but then proceeds more slowly due to the relatively long half-life of the adipocytes.

**Figure 2:**
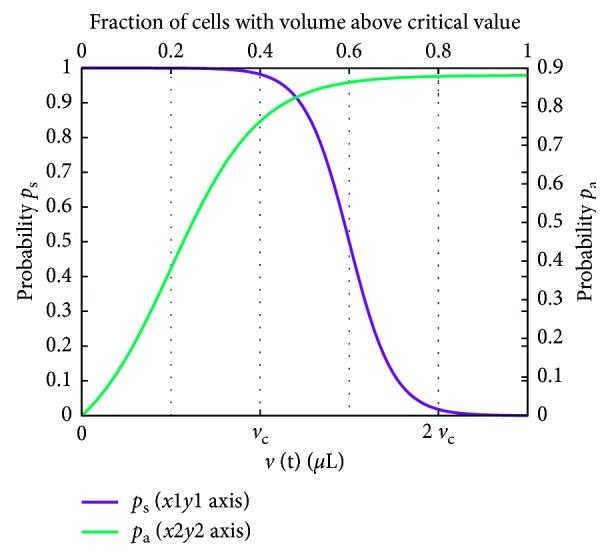
The probability functions *p*_s_ and *p*_a_ corresponding to the following values of the parameters *k*_1_=4, *k*_2_=2, *k*_3_=8 · 10^−6^, *k*_4_=2 · 10^−5^, *k*_5_=5, and *k*_6_=−1. These are the values estimated by using the data in [24, 25] and used throughout the study.

Figure 2 shows *p*_s_ and *p*_a_ corresponding to the values of the parameters estimates with experimental data as described in the following section.

The process described above simulates the weight gain process resulting from a prolonged highly caloric diet described in the following section. To compute the weight from the population of simulated adipocytes, we use equations (3) and (2) and compute the weight changes at time *t* as follows:

(9) $$\begin{array}{c} \text{BW}\left(t\right)-\text{BW}\left(0\right)=\text{FM}\left(0\right)\cdot \frac{1}{1-\delta }\cdot \frac{V\left(t\right)-V_{0}}{V_{0}}\\ =\frac{\text{FM}\left(0\right)}{1-\delta }\left(\frac{\sum_{j=1}^{N\left(t\right)}v_{j}\left(t\right)}{v_{0}N_{0}}-1\right), \end{array}$$

where BW(0) and FM(0) are, respectively, the initial values for body weight and the fat mass, *N*(0)=*N*_0_ is the initial number of adipocytes, *V*_0_=*v*_0_ *N*_0_ is the initial volume of the tissue, *v*_*j*_ is the volume of the *j*th adipocyte, and *V*(*t*)=∑_*j*=1_^*N*(*t*)^ *v*_*j*_(*t*) is the volume of the tissue at time *t*.

### 2.2. Modeling the Link to Inflammation

Macrophages are at heart of many immune-related phenomena including inflammation, but their complexity and plasticity only recently have gained much appreciation. In particular, the differentiation of macrophages into the two phenotypes M1 (pro-) and M2 (anti-inflammatory) is the topic of one of our previous works [11] and has been exploited also in the present study.

The goal of our model is to determine how an excess of calorie triggers, in the long run, a state of low-grade inflammation through the accumulation in the adipose tissue of proinflammatory macrophages [27, 28]. The link from the excess calories to macrophage differentiation to the proinflammatory phenotype is provided by the fact that the increase in volume of adipocytes does not just lead to adipogenesis from precursors but also induces the immune system to set up the inflammatory condition [26, 27]. This process is modeled by having adipocytes which are stimulating adipogenesis (with probability *p*_*a*_) to also secrete inflammatory cytokines such as tumor necrosis factor alpha (TNFa), interleukin-6 (IL-6), and interleukin-1beta (IL-1b). These cytokines create a milieu for the differentiation of macrophages into the M1 (proinflammatory) phenotype as described in [11], thus providing a positive feedback on the further exacerbation of the inflammation.

### 2.3. Available Data and Parameters Estimation

In this section, we describe the data used to find the parameters of the model (*i.e.*, *k*_1_, *k*_2_, *k*_3_, *k*_4_, *k*_5_, and *k*_6_). We run a set of fifty independent simulations with different random number initializations and compute statistics to fit against experimental data from the studies in [24, 25].

The study from Diaz in reference [24] has been conducted for a period of seven months subdivided in five phases: baseline, overfeeding, free diet, underfeeding, and free diet. The only data of interest for us corresponds to the overfeeding phase which went on for 42 days and included 6 lean plus 3 overweighted young men. In this phase, individuals were overfed 50% above their baseline requirement. This corresponds to an excess of 6.2 ± 1.9 MJ/day (1506 kcal/day). The weight increased from 73.7 ± 9.5 kg to 81.4 ± 9.6 kg, namely, 7.6 ± 1.6 kilograms.

In another study [25], Trembley and coauthors have investigated the effect of overfeeding on energy expenditure in 23 young men (21 ± 2 years, 1.74 ± 0.06 meters tall) subjected to a diet consisting of a surplus of caloric intake of 353 MJ. These individuals were overfed 6 days a week for 100 days, that is, 84 days of overfeeding on the total of 100 days. Dietary regimen consisted of 4.2 MJ/day (*i.e.*, 1004 kcal/days) over the preestablished energy cost of weight maintenance. According to this study, the weight of the subjects changed from 60.3 kg (±8.0) to 68.4 kg (±8.2).

**Figure 3:**
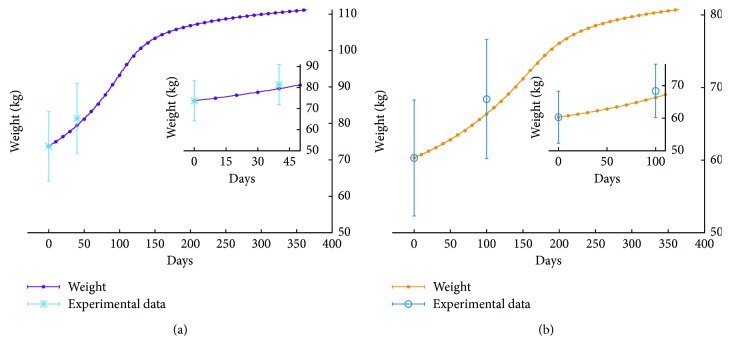
Simulation agreement with the overfeeding phase of the study of (a) Diaz [24], matching an excess caloric intake *E*=1506 kcal/day, and (b) Tremblay [25] equating to *E*=1004 kcal/day. In the first case, we simulate a subject with BMI = 24.06, whereas in the second case, the subject has BMI = 20.38. According to the current classification with respect to the body mass index, both subjects are considered normal.

Figure 3(a) shows data from the study in [24] together with the average value of the total weight BW(*t*) for fifty runs corresponding to an excess of *E*=1506 kcal/day for the whole duration of the simulated period of a year. In these runs, we simulate a 36-year-old male individual 175 cm tall weighting 73.7 kg. The simulation curve is in accordance with the experimental data.

For the second set of data, those of the study in [25], we have performed simulations of a high calorie intake corresponding to an excess of 1004 kcal/day for 6 days a week, for a 36-year-old 60 kg male subject 172 cm tall.  Figure 3(b) shows that the average weight computed on fifty independent runs is also in accordance with this set of experimental data.

## 3. Results and Discussion

To have a comprehensive view, simulations have been performed for diverse calories excess intake (here again each of them were run fifty times to account for stochastic variability).

**Figure 4:**
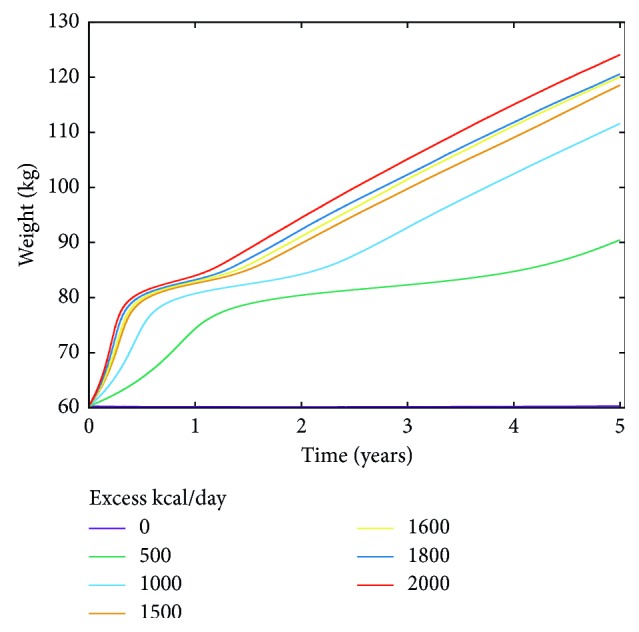
Simulated body weight gain over a period of five years as a function of different excess calorie intake (kcal/day). The simulated individual is a 35-year-old male with an initial BMI of about 21, *i.e.*, a normal subject.

Figure 4 shows the effect of different high calorie diets expressed in kcal/day on the body weight of a simulated 60 kg, 170 cm height, 35-year-old (slim) male. It is worth to note that, overall, BW(*t*) in Figure 4 is nonlinear and has two phases: the first one due to the swelling of the adipocytes present at the initial time, and the second due to the enlargement of the newly recruited adipocytes. This result suggests that the weight gain dynamics has the characteristics of a nonlinear process with a quicker phase followed by a slower progression due to, respectively, swelling and recruitment of new cells; a peculiar dynamics which certainly depends on the individual characteristics such as the distribution and/or metabolic characteristics of the adipocytes in the adipose tissue.

As for the emergence of the inflammation, we recall that when the simulated adipocytes reach the critical volume *v*_c_, they start releasing inflammatory cytokines TNFa, IL-6, and IL-1b with a certain probability *p*_a_. Through the complex machinery of gene regulation, these cytokines induce the differentiation of macrophage precursors into the M1 proinflammatory form. These detailed dynamics have been implemented in the agent-based model in a previous work as a Boolean network describing the activation/inhibition of key genes [11]. The interested reader can also refer to an older example of use of *gene regulatory network modeling* to implement the differentiation rule of an immune cell. In [29], the differentiation of helper T lymphocytes into the two subtypes Th1 and Th2 is carried through the analysis of the dynamics of a Boolean network representing the gene regulatory machinery pertaining the cellular differentiation [29].

**Figure 5:**
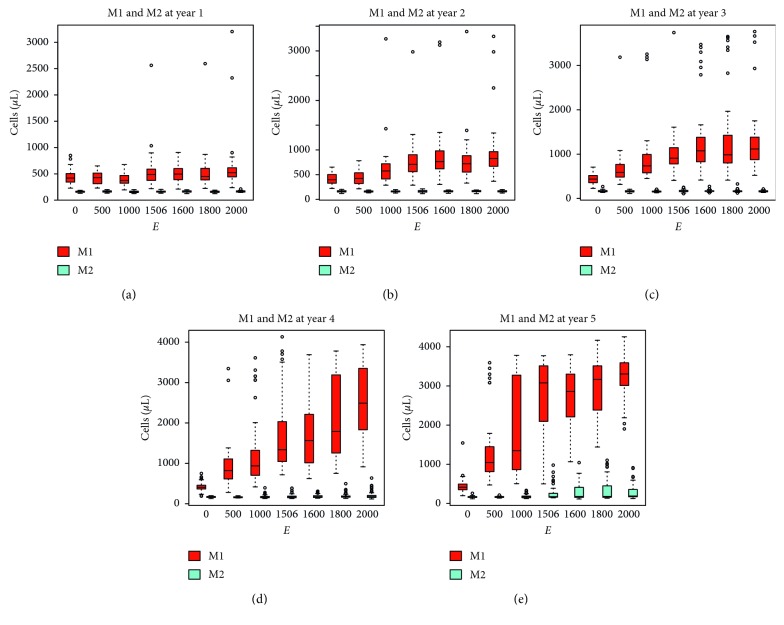
Number of macrophages in the two differentiation classes M1 and M2 per *μ*L of simulated adipose tissue at different time points of simulations of varying calorie diets. The shift toward the M1 proinflammatory phenotype of the macrophage population is not meaningful at the first year (a) but becomes pronounced from year three onwards (c–e) even for relatively lower values of the excess caloric intake (e). Note that at year five (e) also the number of M2 anti-inflammatory macrophages is increased in high calorie diets *E* ≥ 1500, indicating the attempt of the immune system to counterbalance the inflammation by empowering anti-inflammatory mechanisms.

In the present simulations, to show the emergence of an inflammatory state, the population of macrophages is plotted in Figure 5 at year 1 to 5. We observe the presence of M1 proinflammatory macrophages resulting from high levels of proinflammatory cytokines released by adipocytes at first and proinflammatory macrophages subsequently which therefore exerts a positive feedback on the inflammation. In Figure 5, at year five (Figure 5(e)), there seems to be a critical excess calorie intake *E* ~ 10^3^ for which a larger fraction of macrophages participate to the inflammation. Also note that the shift toward the M1 proinflammatory phenotype of the macrophage population as a function of the excess caloric intake *E* is not meaningful at the first year (Figure 5(a)) but becomes pronounced from year three onwards (Figures 5(c)–5(e)) even for relatively lower values of *E*. Also interesting is the fact that, at year five, the number of M2 anti-inflammatory macrophages is somehow increased for *E* ≥ 1500 kcal/day bearing witness of the hopeless anti-inflammatory effort to restore equilibrium (Figure 5(e)).

**Figure 6:**
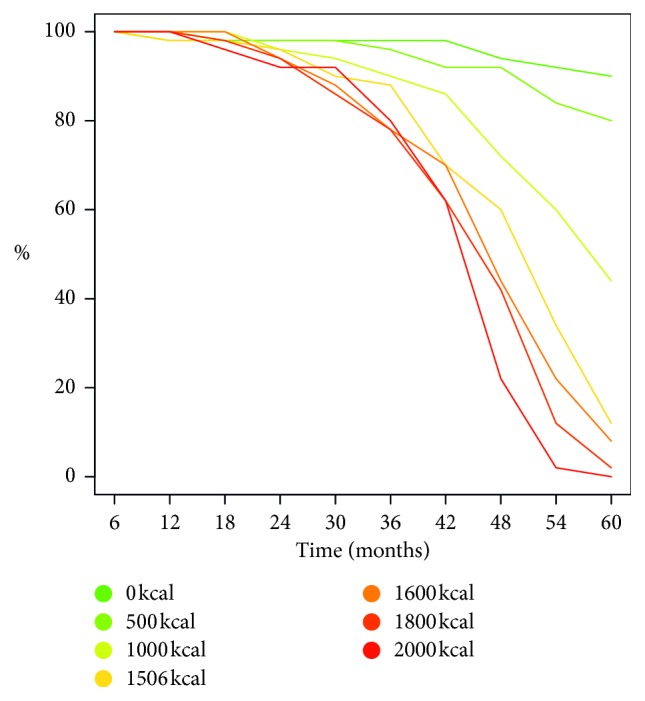
Kaplan–Meier curve of inflammation set-ups as a function of excess calories *E*. The fourth year appears as the tipping point for *E* ≥ 1500 kcal/day.

Another way of looking at the effects of high calorie diets on the setup of the inflammation is by plotting the percentage of cases that did not trigger an inflammatory reaction for each dietary conditions over a time period of five years. These are the Kaplan–Maier curves of Figure 6 pointing to the fourth year as the critical one in the consolidation of the inflammatory state for diets matching *E* ≥ 1500 kcal/day.

## 4. Conclusions

Computational multiscale modeling of immune-related diseases is a growing field of the study [30]. In the present work, we discuss a recently developed computational model for the simulation of the weight gain process leading to inflammation. The model is initialized with some anthropometric measures of the individual such as age, weight, height, and gender, and simulates, on the basis of an excess calorie diet and the accumulation of fat in the adipocyte tissue which is ultimately responsible for the inflammation. The model calculates the body weight as a function of the size and of the volume of the adipocytes which in turn swells and increases in number as a consequence of the lipid accumulation due to the excess of calorie intake.

The parameters of the model have been chosen to reproduce the weight gain increase in the relatively short period (*i.e.*, few months) of two separate experimental studies [24, 25]. By extending the high calorie period to five years, the model shows a weight increase which is within reasonable ranges (Figure 4).

Concurrently to the weight gain, the simulation describes the emergence of the inflammatory state in terms of the relative fraction of macrophages differentiating into the proinflammatory M1 phenotype. As expected, the magnitude of the inflammation correlates with the calorie excess of the diet. Interestingly, it also shows the attempt of the immune system to counterbalance the inflammation eliciting a smaller fraction of anti-inflammatory macrophages M2 (Figure 5(e)).

A number of improvement to this model can be foreseen as, for instance, the effect of physical exercise on the release of the pro/anti-inflammatory cytokine IL-6 [31], a better definition of the individual in terms of not just weight and age but also fitness level and daily energy expenditure [32] or a more detailed transformation of the dietary input into the calorie intake [33]. The latter is the core of a work in progress which will be submitted for publication in due date. As another direction of work, we are planning to extend the model to account for the use of drugs for the treatment of diabetes. This step, which implies a number of complex modifications to account for the impairment of pancreatic beta-cells and the establishment of insulin sensitivity, could enable the model to evaluate treatment schedules not much differently to what was previously done for the optimisation of vaccines [34, 35].

## Data Availability

The data used in this study are from published literature articles and therefore are publicly available.
